# Prevalence of *Chlamydia trachomatis*-Specific Antibodies before and after Mass Drug Administration for Trachoma in Community-Wide Surveys of Four Communities in Nepal

**DOI:** 10.4269/ajtmh.17-0102

**Published:** 2017-11-06

**Authors:** Sarah E. Gwyn, Lingwei Xiang, Ram Prasad Kandel, Deborah Dean, Manoj Gambhir, Diana L. Martin

**Affiliations:** 1 IHRC, Inc. Contractor at the Centers for Disease Control and Prevention, Atlanta, Georgia;; 2 Rollins School of Public Health, Emory University, Atlanta, Georgia;; 3 Lumini Eye Hospital, Bhairahawa, Nepal;; 4 UCSF Benioff Children’s Hospital Oakland, Children’s Hospital Oakland Research Institute, Oakland, California;; 5 University of California at San Francisco and Berkeley Graduate Program in Bioengineering, Berkeley, California;; 6 Department of Epidemiology and Preventive Medicine, Monash University, Melbourne, Australia;; 7 Health Economics and Modeling Unit, National Center for Emerging and Zoonotic Infectious Diseases Centers for Disease Control and Prevention, Atlanta, Georgia;; 8 Division of Parasitic Diseases and Malaria, Centers for Disease Control and Prevention, Atlanta, Georgia

## Abstract

The target end date for the global elimination of trachoma as a public health problem is 2020. As countries begin the process for submitting their dossier for the validation of elimination of trachoma as a public health problem, strategies for post-validation surveillance must be considered. Seroprevalence of antibodies against antigens from the causative bacteria *Chlamydia trachomatis* (*Ct*) in young children has been shown to reflect trachomatous inflammation–follicular (TF) rates in both endemic and previously endemic settings. However, none of these studies has directly compared age seroprevalence in the same communities before and after mass drug administration (MDA) for trachoma. Here we report a marked shift in age seroprevalence curves in four villages in Kapilvastu District, Nepal, before and after MDA. Clinical examinations were performed and blood was taken before (*N* = 659) and 5 years after (*N* = 646) MDA. Rates of TF decreased from 17.6% in ≤ 9-year-olds before MDA (*N* = 52) to 0% in ≤ 9-year-olds (*N* = 73) after MDA. Positive antibody responses to *Ct* in the entire population decreased from 82.1% pre-MDA to 35.8% post-MDA, whereas those among ≤ 9-year-olds decreased from 59.6% to 4.1%. These data show that the postintervention decrease in TF was reflected in a drop in anti-*Ct* antibody responses, suggesting that antibody responses could be useful indicators for post-validation surveillance.

## INTRODUCTION

Trachoma is caused by repeated ocular infection with *Chlamydia trachomatis* (*Ct*) and is the leading infectious cause of blindness in the world today. Over 200 million people are at risk of infection with an estimated 1.9 million people blind or visually impaired due to trachoma (www.trachomacoalition.org/GET2020). Efforts by the World Health Organization (WHO) and other partners are underway to eliminate trachoma as a public health problem by 2020 through implementation of the SAFE strategy: surgery to correct trichiasis (one or more eyelashes rubbing against the globe of the eye), mass drug administration (MDA) of antibiotics for all individuals living in endemic districts, facial cleanliness, and environmental improvement. The Nepali Government Trachoma Program has conducted annual MDA with azithromycin and offered surgery to correct trichiasis in trachoma-endemic regions of Nepal. The MDA program commenced in 2007 and most of the villagers received MDA each year for 3 years.

The current WHO endpoint for MDA is a prevalence of trachomatous inflammation–follicular (TF) of < 5% in children aged 1–9 years district-wide. As MDA programs strive to reduce infection prevalence, and efforts to eliminate trachoma are intensified, post-elimination surveillance will become an increasing programmatic priority and challenge. Funding for TF-based surveillance is unlikely to be a priority in post-elimination settings, and in hypoendemic settings, follicular conjunctivitis may be caused by non-chlamydial bacteria and misdiagnosed as trachoma.^1^ Clinical examinations can also be difficult to standardize across trained staff,^2–5^ and this difficulty will increase as the prevalence of TF decreases in response to elimination program interventions. Monitoring conjunctival infection by commercial nucleic acid amplification tests is an option but is costly and time consuming.^5^ Age-specific prevalence of antibody responses to *Ct* at the community level could provide an informative proxy measure of the intensity of transmission. Serological tests for measuring antibodies represents a means for monitoring cumulative exposure to ocular infection in children.^6–10^ Serological testing would also facilitate integration of trachoma surveillance with other health program activities in which blood collection is occurring, such as demographic and health surveys, malaria indicator surveys, or vaccine-coverage surveys.

Here, we examined the use of serological tools for monitoring and evaluation in post-MDA settings by assessing the age-specific prevalence of clinical signs and antibody responses of trachoma within four communities that received MDA for trachoma 5 years before the study.

## MATERIALS AND METHODS

### Study population.

The study was conducted in four villages in the Kapilvastu District of the Lumbini Zone in southwestern Nepal. The three annual rounds of MDA were completed in 2009 (given in 2007, 2008, and 2009). All community members aged 1 year and over were invited to participate in the study. Participation rates were 81–87% across the four villages. The same communities were sampled before MDA (2000 and 2002) and after MDA (2014). Demographic data on villages are presented in Supplemental Table 1; two of these villages were contiguous and so treated as a single site in this table.

### Ethics statement.

Institutional Review Board (IRB) approvals were obtained for both data collections from Children’s Hospital and Research Center Oakland (IRB number 2013-043) and by Nepal Netra Jhoti Sangh (Nepali Prevention of Blindness Program), and data were not anonymized for these researchers. Centers for Disease Control and Prevention researchers were non-engaged (i.e., did not have access to patient-identifying information) in the study. All study participants gave written informed consent, or written parental consent was obtained for participants under the age of 18.

### Clinical examination for trachoma.

Grading of the upper tarsus of each study participant was performed according to the WHO simplified trachoma grading system^11^ by experienced graders (D.D. and R.P.K.).

### Blood collection and preparation.

Serum or finger prick blood was obtained from all participants; 2000/2002 samples were all serum and 2014 samples were a mix of finger prick blood from primarily < 15-year-olds and serum from ≥ 15-year-olds. Finger prick blood was collected onto filter papers with six circular extensions calibrated to absorb 10 μL of whole blood (TropBio Pty Ltd, Townsville, Queensland, Australia) and stored at −20°C before use. One bloodspot extension for each participant was eluted overnight at 4°C with 500 μL of phosphate-buffered saline (PBS) containing 0.5% bovine serum albumin (BSA), 0.05% Tween 20, 0.02% sodium azide, 0.5% polyvinyl alcohol, and 0.8% polyvinylpyrrolidone, designated as PBN1. This elution was equivalent to a serum dilution of approximately 1:100. Eluates (100 μL) were diluted to a final volume of 400 μL in PBN1 with 0.5% w/v of *Escherichia coli* crude extract to block nonspecific binding [8] for a final serum dilution of approximately 1:400. Sera were diluted 1:400 in 400 μL PBN1 with 0.5% w/v of *E. coli* crude extract and stored overnight at 4°C.

### Multiplex bead array assay.

Bloodspot eluates and serum dilutions were screened in duplicate with Pgp3 and CT694 antigen-coupled beads in a multiplex bead assay as previously described.^6^ Briefly, Pgp3- and CT694-coupled beads (2,500 each) were added to each well of a prewet filter plate (Millipore, Billerica, MA) and washed twice with 100 μL of 0.05% Tween 20 in PBS (PBST)-. Control sera and bloodspot eluates (1:400) were added in duplicate at 50 μL per well to the beads. The plates were vigorously shaken for 30 seconds, covered, and then shaken at room temperature for 1.5 hours. After incubation, wells were washed three times with PBST with a vacuum device (Millipore). Total immunoglobulin G (IgG) was detected with 50 ng of biotinylated mouse antihuman total IgG (SouthernBiotech, Birmingham, AL) and 40 ng of biotinylated mouse antihuman IgG4 (Invitrogen, South San Francisco, CA) per well in 50 μL PBS containing 0.5% BSA, 0.02% sodium azide, and 0.05% Tween 20 (PBN2). After incubation, wells were washed as mentioned previously. R-phycoerythrin-labeled streptavidin (Invitrogen) was added at a concentration of 250 ng per well and incubated for 30 minutes at room temperature. Wells were washed as previously described, after incubation. Wells were additionally incubated in 50 μL of PBN2 to remove any loosely bound antibodies for 30 minutes with shaking. After the final incubation in PBN2, wells were vacuum-evacuated and washed once with PBST. Beads were suspended in 125 μL PBS, shaken, and immediately read on a BioPlex 200 instrument (Bio-Rad, Hercules, CA) equipped with Bio-Plex Manager 6.0 software (Bio-Rad). The median fluorescence intensity (MFI) for each sample was recorded and background from the blank well subtracted out (MFI-BG). Cutoffs for antibody positivity were determined by receiver operator characteristics curves using non-endemic pediatric samples from Milwaukee (*N* = 116) and polymerase chain reaction positives from Tanzania (*N* = 41). The cutoff in MFI-BG units for Pgp3 was 801 (indeterminate range: 641–961) and the cutoff for CT694 was 383 (indeterminate range 195–571).

### Statistical methods.

All analyses were conducted using GraphPad Prism 6.00 for Windows, GraphPad Software, La Jolla, CA, www.graphpad.com. A Kolmogorov–Smirnov test was run to compare the differences in seroprevalence by age groups for pre-MDA and post-MDA. One-way analysis of variance test with multiple comparisons was carried out on MFI-BG data comparing each corresponding age groups pre- and post-MDA.

## RESULTS

### Enrollment.

In 2000 and 2002, before the introduction of MDA in these communities, 659 individuals (39.7% male) had blood samples taken and clinical examinations performed. The age range was 2–80 years (median 28). In these same communities in 2014, 5 years after cessation of MDA, 646 individuals (37.2% male) had both blood collection and clinical examinations for trachoma. At this time point, the age range was 3–90 years (median 26).

### TF and Ct-specific antibody prevalence pre- and post-MDA.

TF among children ≤ 9 years old pre-MDA was 17.6% (9/52, 95% confidence interval (CI): 9.4–29.7) and dropped to 0% (0/73, 95% CI: 0–5.6) post-MDA (*P* = 0.0002, Table 1). The percentage of individuals ≤ 9 years old with any positive anti-*Ct* response pre-MDA was 59.6%, (31/52, 95% CI: 46.1–71.8) with 59.6% (31/52, 95% CI: 46.1–71.8) recognizing Pgp3 and 53.8% (28/52, 95% CI: 40.3–71.8) recognizing CT694 (Table 1). All ≤ 9-year-olds with anti-CT694 antibodies pre-MDA were also positive for anti-Pgp3 antibodies. After MDA, the percent of individuals ≤ 9 years old with a positive anti-*Ct* response was only 4.1% (3/73, 95% CI: 0–8.4; *P* < 0.0001 pre versus post-MDA), with 2.7% (2/73, 95% CI: 0–6.4) responding to Pgp3 and 1.3% (1/73, 95% CI: 0–3.9) recognizing CT694 (Table 1). None of the ≤ 9-year-olds had antibody responses to both Pgp3 and CT694 post-MDA (Table 1).

**Table 1:** TF prevalence and seroprevalence pre- and post-MDA, Nepal, 2000/2 and 2014

	≤ 9-year-olds		All ages	
	Pre-MDA % (95% CI)	Post-MDA % (95% CI)	Pre-MDA % (95% CI)	Post-MDA % (95% CI)
TF	17.6 (7.25–27.9)	0 (0–5.6)	6.5 (4.6–8.4)	1.24 (0.39–2.09)
Pgp3+	59.6 (46.3–72.9)	2.7 (0.0–6.4)	77.0 (73.6–80.0)	32.0 (28.4–35.6)
CT694+	53.8 (40.3–67.4)	1.3 (0.0–3.9)	71.1 (67.6–74.6)	34.9 (31.22–38.58)
Ab+*	59.6 (46.3–72.9)	4.0 (0.0–8.4)	82.5 (79.6–85.4)	38.6 (34.85–42.35)
Pgp3 + CT694+	53.8 (40.3–67.4)	4.0 (0.0–8.4)	65.5 (61.9–69.1)	26.6 (23.19–30.01)

Data shown are percent positive responses for given indicator, with 95% CI in parentheses for samples taken in 2000/2 (pre-MDA, *N* = 52 for ≤ 9-year-olds and 659 for all ages) and 2014 (post-MDA, *N* = 73 for 1–9-year-olds and 646 for all ages). CI = confidence interval; MDA = mass drug administration; TF = trachomatous inflammation–follicular.

* Denotes positive antibody responses to Pgp3 alone, CT694, alone or both Pgp3 and CT694. Pgp3 + CT694 + denotes positive antibody responses to both antigens.

The MFI of the three ≤ 9-year-olds with positive antibody responses post-MDA was 1,513 and 2,485 for Pgp3 reactivity and 490 for CT694 reactivity (data not shown). By contrast, the specimens from ≤ 9-year-olds taken pre-MDA had median MFI value of 25,160 for positive Pgp3 responses (range 1,200–31,534) and median MFI value of 3,347 for positive CT694 responses (range 460–30,445, data not shown).

When evaluating all ages, pre-MDA TF was 6.5% (43/659, 95% CI: 4.6–8.4) and post-MDA was 1.24% (8/646, 95% CI: 0.39–2.09) (Table 1). The percent of all participants with any positive anti-*Ct* response pre-MDA was 82.5% (544/659, 95% CI 79.6–85.4), with 77.5% (511/659, 95% CI: 73.6–80.0) having antibodies recognizing Pgp3 and 71.1% (469/659, 95% CI: 67.6–74.6) with antibodies recognizing CT694 (Table 1). After MDA, 40.4% (261/646, 95% CI: 36.7–44.2) of individuals had antibody responses to either *Ct* antigen, with 34.9% (226/646, 95% CI: 31.22–38.58) recognizing Pgp3 and 32.0% (207/646, 95% CI: 284–35.6) recognizing CT694 (Table 1).

### Shift in age-specific seroprevalence after MDA.

**Figure 1.**
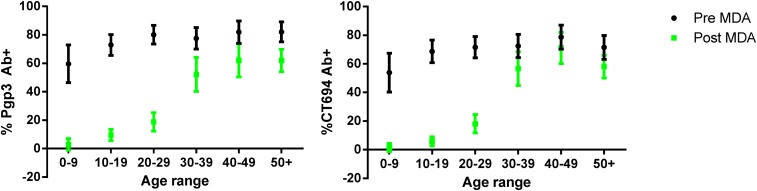
Age seroprevalence pre- and post-MDA, Nepal, 2000/2 and 2014. The percent antibody–positive (*y*-axis) within each age group (*x*-axis, stratified by decade) before (black bars) and after (gray bars) MDA is shown to Pgp3 (left graph) or CT694 (right graph). Vertical bars show 95% confidence intervals. For pre-MDA data *N* = 659 and for post-MDA data *N* = 646. MDA = mass drug administration. This figure appears in color at www.ajtmh.org.

**Figure 2.**
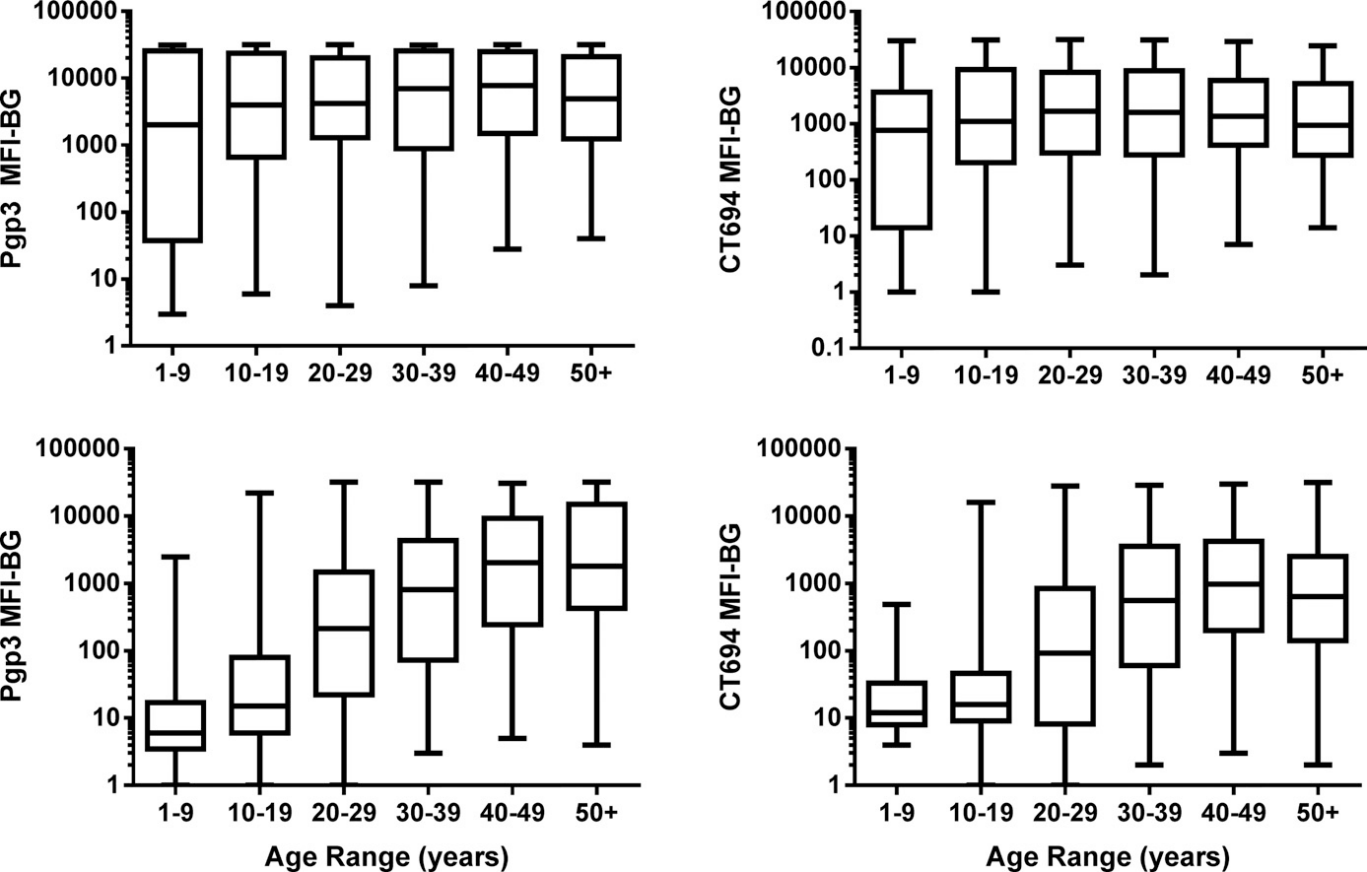
Intensity of anti-*Chlamydia trachomatis* antibody responses by age pre- and post-MDA, Nepal, 2000/2 and 2014. Left: the median fluorescent intensity of anti-Pgp3 antibodies with background subtracted out (Pgp3 MFI-BG) are shown pre-MDA (top) and post-MDA (bottom) for all ages grouped by decade. Right: the MFI of anti-CT694 antibodies with background subtracted out (CT694 MFI-BG) are shown pre-MDA (top) and post-MDA (bottom) for all ages grouped by decade. *Y*-axes are shown on a logarithmic scale. Boxes show the 25–75% quartile of data with solid horizontal lines in the middle representing median proportion-positive for each grouping. The upper and lower whiskers represent the minimum and maximum range. MDI = mass drug administration; MFI = median fluorescence intensity.

Line graphs of seroprevalence grouped by decade of age and age-stratified MFI of the antibody responses differed in the pre- and post-MDA phases (Figures 1 and 2). Pre-MDA, the proportion antibody-positive in all age groups was greater than 60%, and the mean MFI values for the different age groups were statistically similar (Pgp3: *P* = 0.6984; CT694: *P* = 0.5629, Figure 2). Post-MDA, there was a marked age-dependent increase in both the proportion antibody-positive (Figure 1) and the mean MFI values among age groups (Pgp3: *P* < 0.001; CT694: *P* < 0.001, Figure 2).

## DISCUSSION

The data presented here show marked shifts in the *Ct*-specific age–seroprevalence curve in trachoma-endemic communities before and after cessation of MDA. Rates of TF in ≤ 9-year-olds dropped from 17.6% before MDA to 0% after MDA in these communities.

Antibody positivity in ≤ 9-year-olds before MDA was approximately 59%, whereas the TF rates in this age group were only 17.6%. This confirms previously reported data that antibody positivity rates exceed TF rates in trachoma-endemic communities.^6,12^ In multiple studies in trachoma-endemic communities, the antibody positivity rate is approximately 2.5 to 5× that of the TF rate.^6,12^ This suggests that antibody responses are longer-lived than TF responses, which themselves can last for weeks or months.^2^ The relationship between TF and antibody-positive rates in trachoma-endemic communities will be critical for threshold determination for potential programmatic use of seroprevalence assays.

Agreement between antibody responses against Pgp3 and CT694 among ≤ 9-year-olds differed between the pre- and post-MDA setting; the pre-MDA agreement was 90%, whereas the post-MDA agreement was 0%. For the post-MDA samples collected 5 years after cessation of MDA, no TF was seen in ≤ 9-year-olds, and antibody positivity rates were 2.7% for Pgp3, 1.3% for CT694, and 4.1% for either antigen. This lack of concordance between Pgp3 and CT694 reactivity was seen previously in communities in which trachoma has been controlled or eliminated.^7,13^ Pgp3 is a well-defined immunodominant *Ct* antigen that is encoded in the multi-copy *Ct* plasmid and, therefore, should be highly specific for *Ct*, but not other chlamydial, infection.^14–17^ Available data suggest that CT694 is recognized by the immune system in a smaller proportion of individuals with active urogenital *Ct* infection than Pgp3.^16,18^ The intensities of the responses against Pgp3 and CT694 were also much lower post-MDA than before MDA when transmission was active, possibly related to less exposure to ocular *Ct* in the post-MDA setting. Although Pgp3-positive individuals had low-positive responses, the CT694-positive response post-MDA were in the indeterminate range of the assay, similar to previous observations in Tanzania.^7^ It may be useful to focus operational research on Pgp3, and in fact we have selected Pgp3 as the antigen for use in rapid antibody tests for trachoma.^19^

One explanation for this shift in the age–seroprevalence data in 2014 is that other factors during this time frame ca. 1999 caused a decrease in transmission before the initiation of MDA in 2007. One potential factor is the improved socioeconomic status of the community that occurred as young Nepali men worked abroad and sent funds home to support their families. If the transmission rates were changing in 1999, those changes might not be reflected in the antibody data from the earlier surveys in 2000 and 2002 as it would take time for any socioeconomic effect to occur. An alternative explanation is that after 5–8 years of low to no transmission after MDA, ocular *Ct* infection in the community antibody responses were waning and that individuals were seroreverting. The only published data on the longevity of the antibody response for trachoma is from a single Tanzanian hyperendemic community (46% TF in 1–6-year-olds) 6 months after MDA, in which no seroreversion was observed.^12^ Despite this, it is not unreasonable to predict that in a setting with a lower initial TF prevalence (17.6% in 1–9-year-olds) that some individuals of all ages would lose their antibody response over time and that repeated exposure may be required to maintain long-lived antibody production for trachoma. There may be some threshold level or number of infections that results in long-lived, or possibly irreversible, anti-*Ct* antibody responses. The longevity of the antibody response will determine how restrictive the age range for testing will need to be for post-validation surveillance. In the case of very long-lived antibody responses at a population level, the focus for monitoring the impact of MDA would need to be on children born after the cessation of MDA. If the responses are shorter-lived (months to a few years, rather than decades) and seroreversion occurs in most or all of the population, a broader age range could be considered for monitoring for reemergence of transmission.

A major limitation of this study was the small number of ≤ 9-year-olds enrolled and the lack of 1-year-olds. The WHO indicator age range for TF is 1–9-years and data from children in that range are, therefore, of key importance in studies evaluating the utility of serological testing for trachoma surveillance. One-year-olds routinely have the lowest antibody positivity rates in studies from trachoma-endemic villages,^6,8,12^ so the lack of 1-year-olds here may slightly inflate the pre-MDA seroprevalence estimates but likely have no effect on the post-MDA estimates. Despite the small number of samples from ≤ 9-year-olds in this study (52 pre-MDA and 73 post-MDA), we observed a patently steep drop in antibody positivity among this group post-MDA. However, the number of samples may limit the generalizability of these data in helping to calculate a threshold for antibody positivity for trachoma post-MDA surveillance. In addition, although the setting is characterized as “post-MDA,” there has been considerable socioeconomic improvement in these communities since the “pre-MDA” time frame. Therefore, the stark change in the age–seroprevalence curve may not be attributable solely to MDA. The cause of the decline in TF in these communities may be less important than the fact that the drop in TF prevalence was accompanied by a decrease in antibody prevalence at the 2014 data collection. Finally, although we collected data from four villages, two of these were contiguous and could not be considered as separate clusters, and we therefore could not perform cluster-level analysis without the variance calculations being overwhelmed by the limited number of clusters. This study took advantage of the presence of stored samples from a pre-MDA study in these villages; prospective studies evaluating the pre- and post-MDA seroprevalence in a population-based cluster survey are needed.

These data are the first to directly compare age–seroprevalence data before and after completion of MDA for trachoma from the same communities. We observed a clear and dramatic shift in age–seroprevalence data after intervention, reflecting the TF prevalence before and after MDA. Therefore, serosurveillance for trachoma has the potential as a useful tool for evaluating transmission status in settings that have moved from programs focused on eliminating trachoma as a public health problem to programs with the aim of control.

## Supplementary Material

Supplemental Table.

## References

[1] Burton MJ Hu VH Massae P Burr SE Chevallier C Afwamba IA Courtright P Weiss HA Mabey DC Bailey RL, 2011 What is causing active trachoma? The role of nonchlamydial bacterial pathogens in a low prevalence setting. Invest Ophthalmol Vis Sci 52: 6012–6017. 21693601 10.1167/iovs.11-7326 PMC3176035

[2] Bailey R Duong T Carpenter R Whittle H Mabey D, 1999 The duration of human ocular *Chlamydia trachomatis* infection is age dependent. Epidemiol Infect 123: 479–486. 10694161 10.1017/s0950268899003076 PMC2810784

[3] See CW Alemayehu W Melese M Zhou Z Porco TC Shiboski S Gaynor BD Eng J Keenan JD Lietman TM, 2011 How reliable are tests for trachoma?–a latent class approach. Invest Ophthalmol Vis Sci 52: 6133–6137. 21685340 10.1167/iovs.11-7419 PMC3176003

[4] Solomon AW Peeling RW Foster A Mabey DC, 2004 Diagnosis and assessment of trachoma. Clin Microbiol Rev 17: 982–1011 15489358 10.1128/CMR.17.4.982-1011.2004 PMC523557

[5] Wright HR Taylor HR, 2005 Clinical examination and laboratory tests for estimation of trachoma prevalence in a remote setting: what are they really telling us? Lancet Infect Dis 5: 313–320. 15854887 10.1016/S1473-3099(05)70116-X

[6] Goodhew EB Priest JW Moss DM Zhong G Munoz B Mkocha H Martin DL West SK Gaydos C Lammie PJ, 2012 CT694 and pgp3 as serological tools for monitoring trachoma programs. PLoS Negl Trop Dis 6: e1873. 23133684 10.1371/journal.pntd.0001873 PMC3486877

[7] Martin DL 2015 Serology for trachoma surveillance after cessation of mass drug administration. PLoS Negl Trop Dis 9: e0003555. 25714363 10.1371/journal.pntd.0003555 PMC4340913

[8] Martin DL 2015 Serological measures of trachoma transmission intensity. Sci Rep 5: 18532. 26687891 10.1038/srep18532 PMC4685243

[9] West SK Munoz B Weaver J Mrango Z Dize L Gaydos C Quinn TC Martin DL, 2016 Can we use antibodies to *Chlamydia trachomatis* as a surveillance tool for National Trachoma Control Programs? Results from a District Survey. PLoS Negl Trop Dis 10: e0004352. 26771906 10.1371/journal.pntd.0004352 PMC4714879

[10] Zambrano AI Sharma S Crowley K Dize L Munoz BE Mishra SK Rotondo LA Gaydos CA West SK, 2016 The World Health Organization recommendations for trachoma surveillance, experience in Nepal and added benefit of testing for antibodies to *Chlamydia trachomatis* pgp3 protein: NESTS Study. PLoS Negl Trop Dis 10: e0005003. 27654497 10.1371/journal.pntd.0005003 PMC5031451

[11] Thylefors B Dawson CR Jones BR West SK Taylor HR, 1987 A simple system for the assessment of trachoma and its complications. Bull World Health Organ 65: 477–483. 3500800 PMC2491032

[12] Goodhew EB 2014 Longitudinal analysis of antibody responses to trachoma antigens before and after mass drug administration. BMC Infect Dis 14: 216. 24755001 10.1186/1471-2334-14-216 PMC4016634

[13] Pant BP 2016 Control of trachoma from Achham district, Nepal: a cross-sectional study from the Nepal National Trachoma Program. PLoS Negl Trop Dis 10: e0004462. 26871898 10.1371/journal.pntd.0004462 PMC4752456

[14] Chen D Lei L Lu C Galaleldeen A Hart PJ Zhong G, 2010 Characterization of Pgp3, a *Chlamydia trachomatis* plasmid-encoded immunodominant antigen. J Bacteriol 192: 6017–6024. 20851898 10.1128/JB.00847-10 PMC2976438

[15] Comanducci M Manetti R Bini L Santucci A Pallini V Cevenini R Sueur JM Orfila J Ratti G, 1994 Humoral immune response to plasmid protein pgp3 in patients with *Chlamydia trachomatis* infection. Infect Immun 62: 5491–5497. 7960130 10.1128/iai.62.12.5491-5497.1994 PMC303293

[16] Wang J Zhang Y Lu C Lei L Yu P Zhong G, 2010 A genome-wide profiling of the humoral immune response to *Chlamydia trachomatis* infection reveals vaccine candidate antigens expressed in humans. J Immunol 185: 1670–1680. 20581152 10.4049/jimmunol.1001240

[17] Wills GS Horner PJ Reynolds R Johnson AM Muir DA Brown DW Winston A Broadbent AJ Parker D McClure MO, 2009 Pgp3 antibody enzyme-linked immunosorbent assay, a sensitive and specific assay for seroepidemiological analysis of *Chlamydia trachomatis* infection. Clin Vaccine Immunol 16: 835–843. 19357314 10.1128/CVI.00021-09 PMC2691054

[18] Gao XB Xiao M Wang J Liu YJ Liu QZ Qi ML, 2015 Optimization of candidate proteins for serological screening of *Chlamydia trachomatis* infection. Genet Mol Res 14: 12240–12246. 26505372 10.4238/2015.October.9.12

[19] Gwyn S Mitchell A Dean D Mkocha H Handali S Martin DL, 2016 Lateral flow-based antibody testing for *Chlamydia trachomatis*. J Immunol Methods 435: 27–31. 27208400 10.1016/j.jim.2016.05.008

