# Supplementary material for: Prevalence of Chlamydia trachomatis-Specific Antibodies before and after Mass Drug Administration for Trachoma in Community-Wide Surveys of Four Communities in Nepal
Source: Am J Trop Med Hyg. 2017 Nov 6;98(1):216–20. doi: 10.4269/ajtmh.17-0102 (PMC5928690; doi:10.4269/ajtmh.17-0102)
Supplement: Supplementary file 1 [file tpmd170102.SD1.pdf]

Supplemental Table 1

Demographic data stratified by village

Pre-MDA	*N*	Age range	Median age	Female	Male
Village 1	194	7–75	25.5	113 (58.2%)	81 (41.7%)
Village 2	243	5–80	30	150 (61.7%)	93 (38.2%)
Villages 3/4	222	2–75	25.5	134 (60.4%)	88 (39.6%)

Post MDA	*N*	Age range	Median age	Female	Male
Village 1	224	3–85	15.0	143 (63.8%)	81 (36.2%)
Village 2	118	3–88	34.5	68 (57.6%)	50 (42.4%)
Villages 3/4	304	3–90	25.0	170 (55.9%)	134 (44.1%)

Villages 3 and 4 were contiguous and are presented as a single site. MDA = mass drug administration; *N* = number of individuals enrolled in each village/site.
